# Self-reported impulsivity in women with borderline personality disorder: the role of childhood maltreatment severity and emotion regulation difficulties

**DOI:** 10.1186/s40479-019-0101-8

**Published:** 2019-03-05

**Authors:** Annegret Krause-Utz, Ezgi Erol, Athina V. Brousianou, Sylvia Cackowski, Christian Paret, Gabriele Ende, Bernet Elzinga

**Affiliations:** 1 Department of Clinical Psychology, Institute of Psychology, Leiden, The Netherlands; 2 Leiden Institute for Brain and Cognition (LIBC), Leiden, the Netherlands; 3 Institute of Psychiatric and Psychosomatic Psychotherapy, Central Institute of Mental Health, Mannheim, Germany; 4 Medical Faculty, University of Heidelberg, Mannheim, Germany; 5 Department of Psychosomatic Medicine and Psychtherapy, Central Institute of Mental Health (CIMH), Mannheim, Germany; 6 Department Neuoimaging, Central Institute of Mental Health (CIMH), Mannheim, Germany

**Keywords:** Abuse and neglect, Borderline personality disorder, Emotion dysregulation, Impulsivity, Childhood trauma

## Abstract

**Background:**

Childhood maltreatment, such as severe emotional, physical, and sexual abuse and neglect, has been linked to impulse control problems and dysfunctional emotional coping. In borderline personality disorder (BPD), a history of childhood maltreatment may worsen difficulties in emotion regulation, which may in turn give rise to impulsive behaviours. The aim of this self-report study was to investigate associations between childhood maltreatment severity, emotion regulation difficulties, and impulsivity in women with BPD compared to healthy and clinical controls.

**Methods:**

Sixty-one female patients with BPD, 57 clinical controls (CC, women with Attention Deficit Hyperactivity Disorder and/or Substance Use Disorder, without BPD), and 60 female healthy controls (HC) completed self-report scales on childhood trauma (Childhood Trauma Questionnaire, CTQ), difficulties in emotion regulation (Difficulties in Emotion Regulation Scale, DERS), and impulsivity (UPPS Impulsive Behaviour Scale). A conditional process analysis was performed to investigate whether emotion dysregulation statistically mediated the effect of childhood maltreatment severity on impulsivity depending on group (BPD vs. CC vs. HC).

**Results:**

Childhood maltreatment, particularly emotional maltreatment, was positively associated with impulsivity and emotion regulation difficulties across all groups. Difficulties in emotion regulation statistically mediated the effect of childhood maltreatment on impulsivity in BPD, but not in the other groups.

**Conclusion:**

In the context of current conceptualizations of BPD and previous research, findings suggest that problems with emotion regulation may be related to a history of childhood maltreatment, which may in turn enhance impulsivity. Targeting emotion dysregulation in psychotherapy and discussing it in relation to childhood maltreatment can help decreasing impulsive behaviors in individuals with BPD. Given the correlational design of our study which does not allow causal conclusions, future studies have to employ prospective, experimental designs and include larger sample sizes to corroborate associations between childhood maltreatment, emotion dysregulation, and impulsivity.

**Electronic supplementary material:**

The online version of this article (10.1186/s40479-019-0101-8) contains supplementary material, which is available to authorized users.

## Introduction

Borderline personality disorder (BPD) is a severe mental disorder, characterized by a pervasive pattern of instability in affect, cognition (i.e., self-image), interpersonal relationships, and impulsive behaviour [1].

Impulsivity and emotion dysregulation are core features of BPD [1–3]. Impulsivity in BPD can have devastating consequences, being closely linked to risky, (para)suicidal behaviour and to difficulties establishing and maintaining stable meaningful relationships [4–7]. Typical expressions of impulsivity in individuals with the disorder include substance abuse, spending sprees, gambling, reckless driving, risky sexual behaviour, sudden relationship break-ups (e.g., treatment dropout), and non-suicidal self-injury (NSSI, e.g., cutting or burning) [3–6, 8]. These impulsive behaviours mainly occur under emotional stress [1, 3, 9–13]. Thus, impulsivity in BPD has been conceptualized as a consequence of malfunctioning emotion regulation mechanisms [2] or even as a “facet of emotional dysregulation” (Sebastian, Jacob, Lieb, & Tüscher, p. 339) [3] rather than an expression of impulsivity as a primary trait.

One risk factor for the development of BPD is severe childhood maltreatment, such as emotional, physical, and sexual abuse, and neglect [2, 14–21]. Current conceptualizations of BPD propose that an interplay of genetic, neurobiological dispositions (e.g., increased affective sensitivity and reactivity) and stressful / traumatic life events hinders the acquisition of functional/adaptive emotional coping mechanisms, resulting in a pervasive form of emotion dysregulation, which is believed to be at the core of the disorder [7, 27]. The biosocial therory by Linehan [27] particularly emphasizes the role of an invalidating (e.g., abusive, neglectful, unstable) environment in the development of emotion dysregulation and impulsivity [2].

Specifically, this theory proposes that difficulties in emotion regulation, stemming from childhood adversities, lead to an increased use of impulsive coping strategies that help down-regulating negative emotions, i.e., that impulsivity is mainly occurring as a response to stress [2]. In line with this, there is growing evidence that deficits in impulse control (e.g., response inhibition) in BPD are substantially modulated by negative, individually salient emotions and primarily occur under stress [9–13].

A remaining research question is whether the effect of childhood maltreatment on impulsivity is mediated by emotion dysregulation and whether this is specific to BPD since emotion dysregulation and impulsivity are also core features of other mental disorders that frequently co-occur with BPD, e.g., ADHD and substance use disorder.

In general, severe childhood maltreatment can have devastating consequences on the development of self-control capacities, i.e., the regulation of impulses and emotions [22–26]. Throughout infancy, childhood, and adolescence, emotions and emotion regulation play an important role in psychosocial development [24]. Early caregiver interactions are essential in shaping healthy emotion regulatory processes, such as adaptations to changes in the environment and other social-cognitive demands [24, 25]. Children exposed to early adverse experiences are at increased risk for developing mood and anxiety disorders, probably due to changes in neurobiological systems involved in the regulation of stress and emotion, e.g., increased stress responsiveness [23]. This can have detrimental consequences across various life domains, since inhibiting strong emotions is crucial to maintaining goal-directed behaviour and self-control [26].

As mentioned above, emotion dysregulation and impulsivity are also core features of other mental disorders that frequently co-occur with BPD, such as ADHD [13, 28–30] and substance use disorder [30, 31]. These disorders are also associated with higher rates of childhood trauma, compared to healthy samples [31–36]. Difficulties in emotion regulation were found to statistically mediate the relationship between childhood trauma severity and substance abuse related impulsivity (e.g., problems controlling cravings) [37, 38]. Likewise, non-acceptance of emotions [39] and not being able to label emotions [40] were related to impulse control problems (e.g., relapses) in problem drinkers and higher substance use rates [41, 42]. Although comorbidity between these disorders and BPD is high [31, 36, 43], not all of these studies controlled for the presence of BPD which may have confounded results.

In summary, evidence suggests that childhood maltreatment is linked to difficulties in emotion regulation and impulsivity, which put individuals at higher risk for developing various psychopathologies. It is not yet entirely clear whether the effect of childhood maltreatment severity on impulsivity is statistically mediated by emotion dysregulation and whether this is more pronounced in BPD, as compared to other clinical samples. Investigating this relationship might help enhancing the understanding of impulsivity in BPD. As a first step in this direction, the present study made use of self-report data to examine the role of emotion dysregulation in the relation between childhood maltreatment and impulsivity in women with BPD compared to healthy controls and clinical controls without BPD.

Given that impulsivity is a complex heterogeneous construct [29, 30, 44], impulsive behaviour was operationalized based on the UPPS Impulsive Behaviour model by Whiteside and Lynam [44]. This model conceptualizes impulsive behaviour as comprising multiple facets: 1) a *lack of premeditation* (difficulties considering the consequences of an action and making accurate plans or precautions), 2) increased *sensation seeking* (tendency to pursue exciting activities, openness to try new potentially dangerous experiences), 3) *lack of perseverance* (maintaining task-related attention and goal-directed behaviour in demanding situations), and 4) *urgency* (tendency to act without forethought during emotional states) [44, 45].

Based on that, the present self-report study investigated whether: 1) higher childhood maltreatment severity predicts higher impulsivity, 2) difficulties in emotion regulation statistically mediate the relationship between childhood maltreatment severity and impulsivity, and 3) this mediating relationship is particularly strong in patients with BPD, as compared to clinical controls (CC, without BPD) as well as female healthy controls.

We hypothesized that, across all participants, childhood maltreatment would positively predict emotion regulation difficulties and impulsivity. We further expected that this mediating relationship would be stronger in BPD patients compared to the other groups.

## Methods

### Participants

*N* = 181 women participated. General inclusion criteria were age between 18 and 46, sufficient language proficiency, and female gender. Recruitment took place at two sites: 1) the Central Institute of Mental Health (CIMH) in Mannheim, Germany, and 2) Leiden University, the Netherlands.

Patients in the BPD group (*n* = 61) were all recruited at the CIMH, in the context of two larger projects designed to investigate impulsivity and provoked aggression [28, 46]. Inclusion criterion for this group was meeting criteria for BPD according to DSM-IV [47]. Exclusion criteria were substance abuse disorder, diagnosis of Attention Deficit Hyperactivity Disorder (ADHD), lifetime history of bipolar-I affective disorder and psychotic disorder, current suicidal crisis, mental deficiency, developmental disorder, and psychotropic medication within 2 weeks prior to the study. Since participants also participated in neuroimaging research, further exclusion criteria were pregnancy and magnetic-resonance-imaging related criteria (brain injury, metal implants, left-handedness, claustrophobia). To ensure that BPD diagnosis was met and to rule out other diagnoses, interested participants were invited for an extensive diagnostic screening and intake session, including the International Personality Disorder Examination (IPDE) [48] and the Structured Clinical Interview for DSM-IV Axis-I (SCID I) [49], as described in more detail below. To assess/exclude adult ADHD diagnosis, the Wender-Reimherr Adult Attention Deficit Disorder Scale and checklists on ADHD symptoms in childhood and adulthood ([“Homburger ADHS-Skalen für Erwachsene”], HASE) [50] were used (see below).

Healthy controls (HC, *n* = 60) were recruited via both sites (CIMH, Leiden University). They were included if they did not have a lifetime history of mental disorders, based on the SCID and the IPDE [48, 49]. Exclusion criteria were severe somatic/neurological disorders and drug use. In addition to the SCID and IPDE, healthy controls further completed the BSL23, WURS-k; ADHD-CL, CAARS, and WRI.

Patients in the CC group (*n* = 57) were partly recruited at the CIMH and partly at Leiden University. Recruitment took place via the outpatient unit of the Department of Psychiatry at the Central Institute of Mental Health (CIMH) in Mannheim, Germany, internet platforms, and/or the research participation website of Leiden University. In Leiden, the recruitment was targeted at individuals experiencing impulse control problems related to substance (drug/alcohol) abuse. The SCID, IPDE, and Mini-International Neuropsychiatric Interview (MINI) respectively [51–53] were used for screening and diagnostic purposes. Exclusion criteria for this CC group was having a diagnosis of BPD as well as scoring higher than Mean = 1 (15th percentile rank) on the Borderline Symptom List 23 Behaviour Checklist [54].

Major diagnoses in the CC group (*n* = 57) were substance use disorder (SUD) (*n* = 29, ~ 50%) and adult ADHD (*n* = 28, ~ 50%, *n* = 3 with comorbid SUD); *n* = 17 (30%) had a comorbid eating disorder, *n* = 27 (47%) had a comorbid anxiety disorder.^1^ The CC group showed similarly high levels of impulsivity and emotion regulation difficulties as the BPD group (see Table 1; for ADHD and SUD separately, see Additional file 2: Table S2, Additional file 3: Table S3 and Additional file 4: Table S4).

**Table 1:** Age, years of education, childhood trauma severity (CTQ sum scores), difficulties in emotion regulation (DERS sum scores), and impulsivity (UPSS Impulsive Behaviour Scale mean scores) in BPD, CC and HC

Variable	BPD (*n* = 61)	CC (*n* = 57)	HC (*n* = 60)	Group statistics (MANOVA)
Age	27.28 ± 6.14 ^a^	30.63 ± 7.63 ^b^	27.28 ± 6.55 ^a^	*F*_*(2, 172)*_ = 4.64, *p* = 0.011, *η*^*2*^_*p*_ = 0.05
Years of Education	10.64 ± 1.80 ^a^	10.63 ± 3.13 ^a^	11.47 ± 0.92 ^b^	*F*_*(2, 172)*_ = 2.53, *p* = 0.054, *η*^*2*^_*p*_ = 0.03
CTQ	66.38 ± 22.07 ^a^	42.36 ± 22.07 ^b^	29.67 ± 4.34 ^c^	*F*_*(2, 170)*_ = 84.20, *p* < 0.001, *η*^*2*^_*p*_ = 0. 50
UPPS	2.57 ± 0.40 ^a^	2.65 ± 0.38 ^a^	0.04 ± 0.03 ^b^	*F*_*(2, 164)*_ = 474.22, *p* < 0.001, *η*^*2*^_*p*_ = 0.85
DERS	97.44 ± 2.72 ^a^	91.52 ± 2.80 ^a^	69.62 ± 2.75 ^b^	*F*_*(2, 170)*_ = 28.46, *p* < 0.001, *η*^*2*^_*p*_ = 0.25

Table shows means ± standard deviations of scores and results of the multivariate analysis of variance, with post-hoc Tuckey tests

*BPD* Borderline Personality Disorder (patient group), *HC* Healthy control group, *CC* Clinical Control group, *CTQ* Childhood Trauma Questionnaire, *DERS* Difficulties in Emotion Regulation Scale, *UPPS* UPPS Impulsive Behaviour Scale

Groups with different superscripts (a, b, c) differ significantly at *p* < 0.05, η2p = effect size partial eta square

Both patient groups (BPD, CC) scored significantly higher in impulsivity and emotion regulation difficulties than HC (all *p* < 0.001) (Table 1). Age did not differ significantly between BPD and HC, while patients in the CC were significantly older than participants in the BPD group and HC (*p* < 0.05, see Table 1). Moreover, there was a trend for differences in years of education, with patients in the BPD group and CC group showing lower education than HC (*p* < 0.05, see Table 1). Therefore, age and education were included as statistical covariates in all analyses.

### Measures

#### Diagnostic instruments

##### Diagnostic assessment of DSM-IV axis I disorders

The *Structured Clinical Interview for DSM-IV axis I disorders* (SCID-I) is a semi-structured clinical interview, designed to determine DSM-IV major mental disorders, administered by trained mental health professionals. It comprises separate modules corresponding to major categories of DSM-IV diagnoses; symptoms are coded as present, subthreshold, or absent based on diagnostic algorithms. Good internal consistency, and moderate to excellent inter-rater reliability of the axis I disorders were reported [49]. The *Mini-International Neuropsychiatric Interview (MINI)* is a well-established screening tool and semi-structured interview developed for systematic diagnostic assessment of mental disorders [51]. The MINI is based on DSM-IV criteria for 17 axis I disorders. It has been found to show very good inter-rater reliability (α > .79), good test-retest reliability (α > .63), high concordance rate with other structured interviews, high patient acceptance, and very good specificity and sensitivity [52, 53].

##### SUD assessment (SCID-I, MINI)

The section on Substance Use Disorder was introduced by the question “Have you ever used alcohol or taken any drugs more than once to get high, to feel better or to change your mood?”. From an indicated drug category, symptoms within the past 12 month were explored (tolerance effects; withdrawal symptoms; ending up taking more drugs than attempted; failure reducing or stop taking drug, spending substantial time (> 2 h) on obtaining, using or in recovering from drug; social, financial, legal, health and/or mental problems, e.g., from being intoxicated, high or hungover while having to fulfil responsibilities at school, at work or at home).

##### BPD assessment

The *International Personality Disorder Examination* (*IPDE*) is a semi-structured clinical interview based on the International Classification of Diseases (ICD 10) and the DSM-III-R classification systems. Reasonably good interrater reliability and temporal stability after an interval of 6 months were reported [48]. In the current study, the IPDE was administered by trained clinicians, interrater reliability was κ =0.77. The *Borderline Symptom List* (BSL-23) is a self-report measure used to assess BPD symptom severity in the past week. Twenty-three statements, such as “I hated myself” and “I thought of hurting myself” are rated on a 5-point Likert scale (0 = *not at all* to 4 = *very strong*). In addition, behavioural aspects related to BPD symptom severity (e.g., NSSI) in the past week are assessed. This scale previously showed high test-retest reliability (*r* = 0.82) [54]. Previously, Cronbach’s *a* for BSL-23 were found to be between 0.94 and 0.97, denoting a high internal consistency. In the current study, Cronbach’s *a* for the BSL-23 was excellent (α =0.97).

*ADHD assessment (“Homburger ADHS-Skalen für Erwachsene”, HASE)* [50]*.* The short version of the *Wender Utah Rating Scale* (*WURS-k*) is a self-report scale consisting of 25 items which retrospectively assess ADHD symptoms in childhood. Items are answered on a five-point Likert scale (0 = “not applicable” to 4 = “applicable”). The *Connor Adult ADHD Rating Scale* (*CAARS*) and the *ADHD-Checklist* (*ADHD-CL*) were used to assesses symptoms of adult ADHD, based on the DSM-IV criteria for ADHD in adulthood [47]. The 66 items of the CAARS are rated on a 4-point Likert scale (0 = “not applicable” to 3 = “very often”), while the 22 items of the ADHD-CL are answered on a three-point scale (0 = “not applicable” to 2 = “applicable”). To verify/exclude ADHD diagnosis, the Wender-Reimherr Interview (WRI) was used, a clinical interview, based on the Wender Adult Attention Deficit Disorder Scale that is conceptualized for adult ADHD. In the current study, Cronbach’s *a* for all ADHD scales were very good to excellent (WURS-k: *a* = 0.89 CAARS: *a* = 0.98; ADHD-CL: *a* = 0.96;)

#### Primary measures

##### Childhood Trauma Questionnaire (CTQ)

Childhood maltreatment severity was assessed using the CTQ [55–57], a self-report scale with five subscales measuring emotional, sexual, and physical abuse, emotional neglect, and physical neglect (5 items each, overall 25 items, between 1 = “never true” to 5= “very often true”). Higher scores indicate the frequency of abuse experiences. The CTQ has demonstrated good psychometric properties, with test-retest reliability ranging from .79 to .84, internal consistency coefficients between α = .66 and α = .94, and good convergent validity with therapist ratings [55–58]. Cronbach’s alpha in the present study suggested very good internal consistency (emotional abuse: α = .96, physical abuse: α = .84, sexual abuse: α = .97, emotional neglect: α = .95) except from the subscale physical neglect (α = .56).

##### UPPS Impulsive Behavior Scale

The UPPS scale was used to assess multiple facets of impulsivity, based on the Five Factor Model of Personality [44, 45]. The scale consists of 45 items related to the four subscales Urgency (12 items; e.g., “I have trouble resisting my cravings (for food, cigarettes).”, “When I feel bad, I will often do things I later regret in order to make myself feel better now.”), (Lack of) premeditation (11 items; e.g., “I don’t like to start a project until I know exactly how to proceed”, “My thinking is usually careful and purposeful.”), (Lack of) perseverance (10 items, e.g., “I generally like to see things through to the end.”), and Sensation seeking (12 items; e.g., “I generally seek new and exciting experiences and sensations.”). Participants rate each item on a 4-point Likert scale (1 = strongly agree to 4 = strongly disagree). Good psychometric properties have been reported, including high internal consistency (α = .82 to .91) [44, 45, 59, 60]. In order to create a score for impulsivity, items for the UPPS subscales’ (lack of) premeditation’ and ‘(lack of) perseverance’ were reversed, so that higher scores indicated more impulsivity. Cronbach’s alpha in the present study suggested good internal consistency (Premeditation: α = .86, Urgency: α = .89, Sensation Seeking: α = .85) except from the subscale Perseverance (α = .63).

##### Difficulties in Emotion Regulation Scale (DERS)

The DERS was used to assess difficulties in emotion regulation [25]. Within a multidimensional framework, the DERS assesses emotion regulation as being *aware* of current emotional experiences, *understanding* them, being able to *accept* and reflect on these emotions, having a clear idea about how to effectively *regulate* them and how to successfully use effective and mature regulation *strategies* [25]. The DERS consists of 36 items which reflect difficulties within each dimension of emotion regulation: ACCEPTANCE (e.g., “When I’m upset, I feel guilty for feeling that way”), STRATEGIES (e.g., “When I’m upset, I know that I can find a way to eventually feel better.”), GOALS (e.g., “When I’m upset, I have difficulty getting work done”), IMPULSES / CONTROL (e.g., “I experience my emotions as overwhelming and out of control.”), AWARENESS (e.g., “When I’m upset, I believe that my feelings are valid and important.”), and CLARITY (e.g., “I have difficulty making sense out of my feelings”). Items are answered on a 5-point Likert scale (between 1 = almost never and 5 = almost always). Internal consistency of overall DERS score (α = .94) and subscales (α = .80 to .91) are good, and high validity with other emotion regulation scales was reported [61]. In the present scoring version, higher scores on the DERS indicate more difficulties in emotion regulation. Internal consistency of the total scale was α = .84.

### Procedure

The study was approved by the ethical committee of the medical faculty of the University of Heidelberg in Mannheim, Germany as well as by the Psychology Ethics Committee of Leiden University. All participants were informed about the background of the study and provided informed consent, study participation could be terminated at any time point without negative consequences. Participants in the BPD and HC group completed the questionnaires (UPPS Scale, DERS, CTQ), in part as paper-pencil versions, and in part (*n* = 28, 15%) via the online survey software Qualtrics (© 2015, Qualtrics, Provo, UT), which included the scales in randomized order. At the end of the study, all participants were debriefed, thanked for their participation and reimbursed (paid a small fee for their participation, 12 Euro/h).

### Statistical analysis

Software IBM SPSS Statistics 22.0 with a-priori defined α-value of *p* < .05, two-tailed, was used. Prior to all analyses, assumptions of linearity, normality of residuals, homoscedasticity and independence of residuals, and outliers (Cook’s distance, Leverage values) were checked. Two extreme outliers were identified (> 3.5 SD from the mean) and removed from the analysis. Multicollinearity was checked based on VIF and tolerance values.

The hypotheses were tested using the PROCESS macro, based on principles by Hayes and Preacher [62, 63]. Childhood maltreatment severity, represented by CTQ sum scores, was defined as predictor (X variable). Impulsivity (mean UPPS scores) as outcome variable (Y), and difficulties in emotion regulation (DERS sum score) as statistical mediator variable (M). Group (BPD, CC, HC) was conceptualized as conditional moderator variable (W).^2^ We tested both the *direct effect* of childhood maltreatment on impulsivity (path c′) and its *indirect effects* through the mediator variable. Path a corresponds to the effects of the predictor variable on the statistical mediator variable, while testing for interactions with group (IE1). Path b refers to the effect of the mediator variable on the outcome variable, testing for interactions with group (IE2). The model also evaluates interactions of group with the statistical mediator variable, i.e., whether the mediating effect is significantly depending on group (IE3). Age and education were added as covariates. A bootstrapping function based on 5000 samples and a confidence interval of 95% was used to quantify effects. In separate analyses, we tested whether results changed, when testing for the four UPPS subscales separately.

The mediator (DERS total) and the dependent variable (UPPS) were significantly but only moderately moderated (*r* = .360, *p* < .001; for correlations between subscales see Additional file 5: Table S5). Due to a potential conceptual overlap between the UPPS subscale Urgency (tendency to act without forethought during emotional states) and DERS, we repeated the above-mentioned conditional mediation analyses excluding the UPPS Urgency subscale (i.e., only using the other three UPPS subscales). We further tested whether the predictor and mediator variable would interact in predicting the outcome variable, when controlling for group, which would give ground for examining moderation. The interaction effect was not significant (*F*_(1,168)_ = 0.62, *p* = .432).

*Total effects* of childhood maltreatment (without taking effects of the intervening variable and group into account - path c) were tested using multiple regression analyses (MRA)^3^ with UPPS scores as dependent variable, controlling for age and education. In a first step, the sum score on the CTQ was entered as predictor. In case of a significant overall effect, the CTQ subscales instead of the sum score were entered as predictors. Since multicollinearity diagnostics revealed very low tolerance values for the subscales emotional abuse and emotional neglect (< 0.24), means for “emotional maltreatment” (emotional abuse and neglect) and “physical maltreatment” (physical abuse and neglect) were created. Additional multiple linear regression analyses were performed to investigate the *total effects* of different subtypes of childhood maltreatment on DERS and DERS on impulsivity respectively.

## Results

**Fig. 1:**
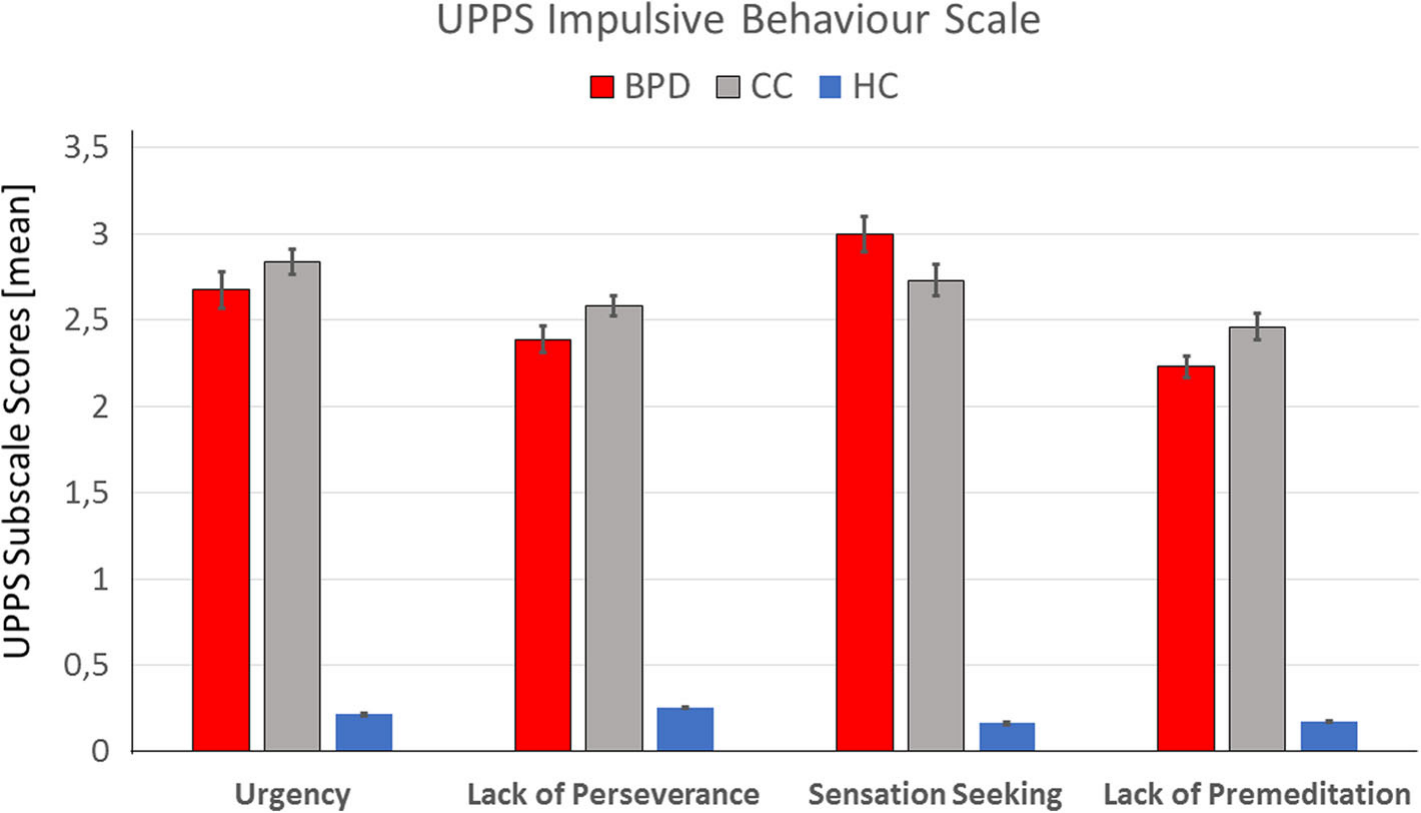
This figure shows means with standard errors of the mean for scores on the UPPS Impulsive Behaviour Scale in patients with Borderline Personality Disorder (BPD), Clinical Controls (CC) and Healthy Controls (HC) as well as results of the MANOVA

**Fig. 2:**
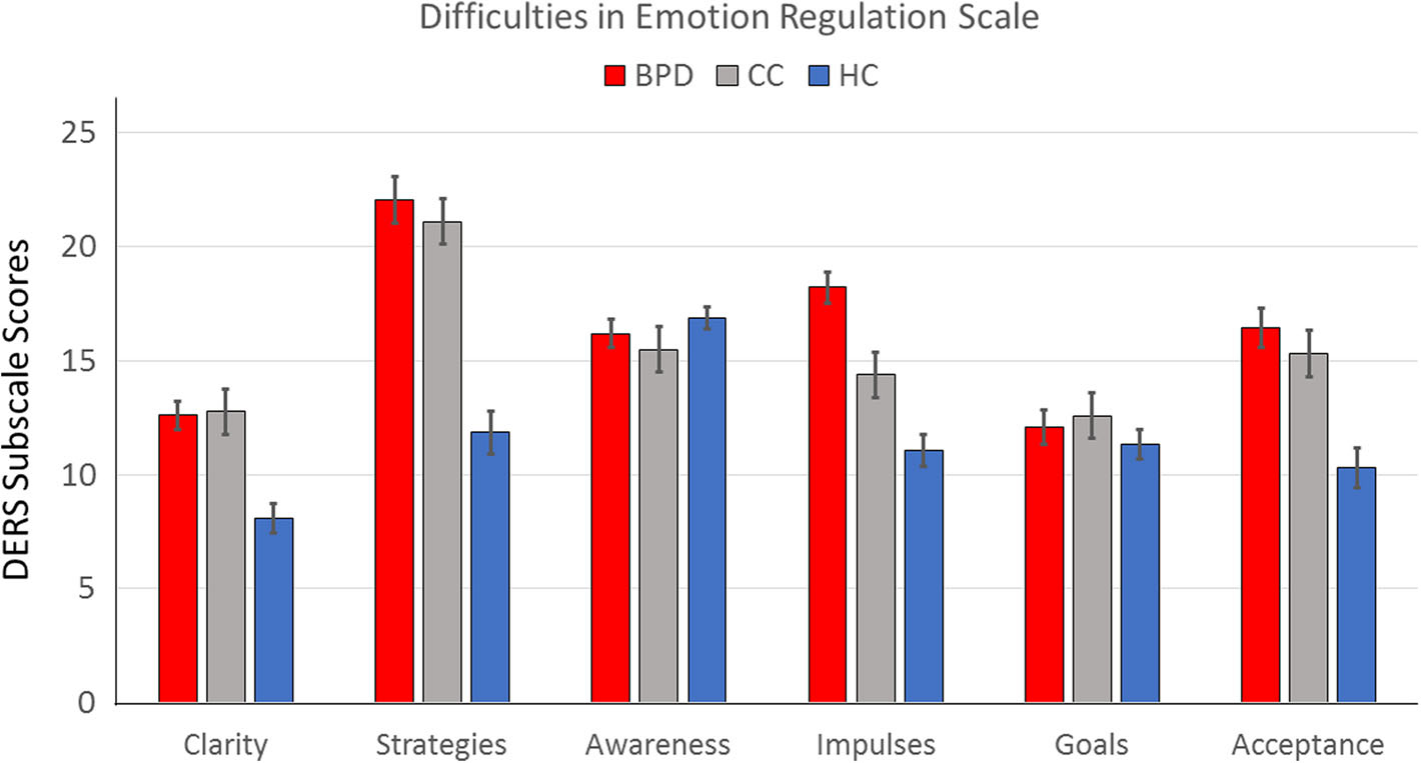
This figure shows means with standard errors of the mean for scores on the Difficulties in Emotion Regulation Scale (DERS) in patients with Borderline Personality Disorder (BPD), Clinical Controls (CC) and Healthy Controls (HC) as well as results of the MANOVA

**Fig. 3:**
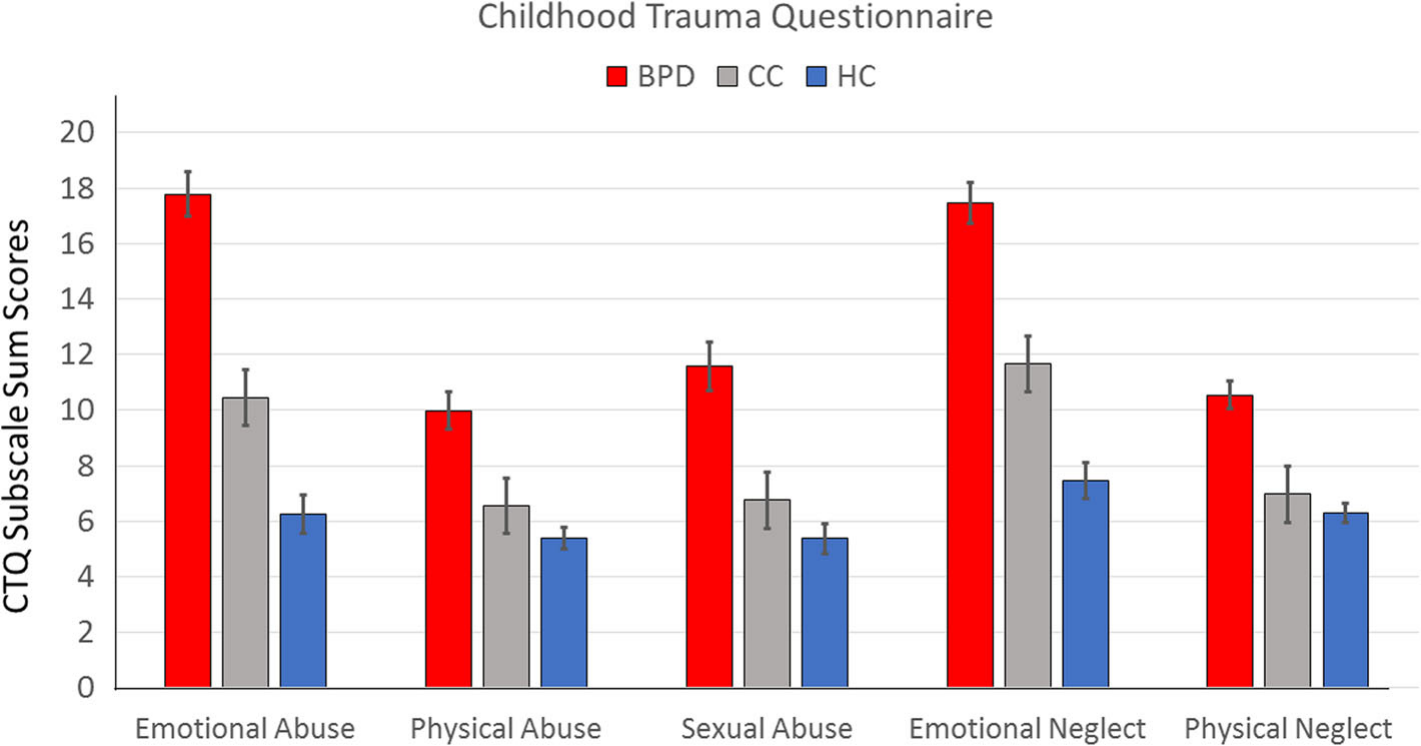
This figure shows means with standard errors of the mean for scores on the Childhood Trauma Questionnaire (CTQ) in patients with Borderline Personality Disorder (BPD), Clinical Controls (CC) and Healthy Controls (HC) as well as results of the MANOVA

Means with standard deviation (SD) for the CTQ, DERS, and UPPS subscales and results of the MANOVAs can be found in Table 2. Patients in the BPD and CC reported higher impulsivity on all UPPS scales than HC, while CC reported significantly higher lack of premeditation than BPD (Fig. 1). On the DERS, the BPD and CC group reported higher lack of clarity, lack of regulation strategies, and more difficulties in accepting emotions than HC, while not differing significantly from each other; levels of emotional awareness and self-perceived goal-directed behaviour were comparable across the three groups (Fig. 2). On the CTQ, BPD patients reported significantly higher levels of emotional abuse and neglect, physical abuse and neglect, and sexual abuse than the other groups; patients in the CC group reported significantly higher levels of emotional maltreatment (abuse and neglect) than HC (Fig. 3).

**Table 2:** Descriptive values for scores on Impulsivity (UPPS Impulsive Behaviour Scale), Emotion Regulation Difficulties (Difficulties in Emotion Regulation Scale) and Childhood Maltreatment Severity (Childhood Trauma Questionnaire) in patients with Borderline Personality Disorder (BPD), Clinical Controls (CC) and Healthy Controls (HC) and results of the MANOVA

Variable	BPD (*n* = 55)	CC (*n* = 54)	HC (*n* = 58)	Group statistics
UPPS Impulsive Behaviour Scale				
UPPS Negative Urgency	2.67 ± 0.87	2.83 ± 0.55	0.22 ± 0.06	*F*_*(2, 164)*_ = 350.27, *p* < 0.001, *η*^*2*^_*(part)*_ = 0.81 *BPD* vs. *CC:* − 0.17 ± 0.11, 95% CI [− 0.43, 0.10] *BPD* vs. *HC:* 2.45 ± 0.11^**^, 95% CI [2.19, 2.72] *CC* vs. *HC:* 2.62 ± 0.11^**^, 95% CI [2.36, 2.88]
UPPS Premeditation	2.23 ± 0.50	2.45 ± 0.55	0.18 ± 0.03	*F*_*(2, 164)*_ = 494.70, *p* < 0.001, *η*^*2*^_*(part)*_ = 0.86 *BPD* vs. *CC:* − 0.22 ± 0.08^*^, 95% CI [− 0.42, − 0.03] *BPD* vs. *HC:* 2.05 ± 0.08^**^, 95% CI [1.86, 2.24] *CC* vs. *HC:* 2.27 ± 0.08^**^, 95% CI [2.09, 2.46]
UPPS Perseverance	2.40 ± 0.64	2.58 ± 0.45	0.25 ± 0.05	*F*_*(2, 164)*_ = 474.22, *p* < 0.001, *η*^*2*^_*(part)*_ = 0.85 *BPD* vs. *CC:* − 0.18 ± 0.09, 95% CI [− 0.38, 0.02] *BPD* vs. *HC:* 2.15 ± 0.08^**^, 95% CI [1.95, 2.34] *CC* vs. *HC:* 2.32 ± 0.08^**^, 95% CI [2.12, 2.52]
UPPS Sensation Seeking	3.02 ± 0.83	2.74 ± 0.69	0.17 ± 0.04	*F*_*(2, 164)*_ = 372.40, *p* < 0.001, *η*^*2*^_*(part)*_ = 0.82 *BPD* vs. *CC:* 0.27 ± 0.12, 95% CI [− 0.01, 0.55] *BPD* vs. *HC:* 2.85 ± 0.12^**^, 95% CI [2.58, 3.12] *CC* vs. *HC:* 2.58 ± 0.12^**^, 95% CI [2.30, 2.85]
Difficulties in Emotion Regulation Scale				
DERS Clarity	12.60 ± 4.81	12.81 ± 4.87	8.12 ± 2.58	*F*_*(2, 164)*_ = 22.66, *p* < 0.001, *η*^*2*^_*(part)*_ = 0.22 *BPD* vs. *CC:* − 0.21 ± 0.80, 95% CI [− 2.12, 1.69] *BPD* vs. *HC:* 4.48 ± 0.79^***^, 95% CI [2.61, 6.35] *CC* vs. *HC:* 4.69 ± 0.79^***^, 95% CI [2.82, 6.57]
DERS Regulation strategies	21.89 ± 8.03	21.30 ± 7.09	11.84 ± 3.80	*F*_*(2, 164)*_ = 42.45, *p* < 0.001, *η*^*2*^_*(part)*_ = 0.34 *BPD* vs. *CC:* 0.59 ± 1.25, 95% CI [− 2.36, 3.55] *BPD* vs. *HC:* 10.05 ± 1.23^***^, 95% CI [7.14, 12.95] *CC* vs. *HC:* 9.45 ± 1.23^***^, 95% CI [6.54, 12.37]
DERS Awareness	16.25 ± 4.96	15.53 ± 3.62	16.88 ± 3.62	*F*_*(2, 164)*_ = 1.49, *p* = 0.23, *η*^*2*^_*(part)*_ = 0.02 *BPD* vs. *CC:* 0.72 ± 0.79, 95% CI [− 1.14, 2.58] *BPD* vs. *HC:* − 0.62 ± 0.77, 95% CI [− 2.45, 1.20] *CC* vs. *HC:* − 1.34 ± 0.78, 95% CI [− 3.18, 0.50]
DERS Control	18.05 ± 5.35	14.41 ± 5.30	11.09 ± 2.81	*F*_*(2, 164)*_ = 32.28, *p* < 0.001, *η*^*2*^_*(part)*_ = 0.28 *BPD* vs. *CC:* 3.65 ± 0.88^***^, 95% CI [1.56, 5.74] *BPD* vs. *HC:* 6.97 ± 0.87^***^, 95% CI [4.92, 9.02] *CC* vs. *HC:* 3.32 ± 0.87^**^, 95% CI [1.26, 5.38]
DERS Goals	12.04 ± 5.66	12.69 ± 4.94	11.33 ± 4.25	*F*_*(2, 164)*_ = 1.04, *p* = 0.35, *η*^*2*^_*(part)*_ = 0.01 *BPD* vs. *CC:* − 0.65 ± 0.95, 95% CI [− 2.90, 1.60] *BPD* vs. *HC:* 0.71 ± 0.94, 95% CI [− 1.51, 2.92] *CC* vs. *HC:* 1.36 ± 0.94, 95% CI [− 0.87, 3.58]
DERS Acceptance	16.35 ± 6.76	15.56 ± 6.52	10.36 ± 3.40	*F*_*(2, 164)*_ = 18.31, *p* < 0.001, *η*^*2*^_*(part)*_ = 0.18 *BPD* vs. *CC:* 0.79 ± 1.10, 95% CI [− 1.80, 3.38] *BPD* vs. *HC:* 5.98 ± 1.08^***^, 95% CI [3.43, 8.53] *CC* vs. *HC:* 5.19 ± 1.08^***^, 95% CI [2.63, 7.75]
Childhood Trauma Questionnaire				
CTQ Emotional Abuse	17.53 ± 6.12	10.46 ± 5.11	5.95 ± 1.28	*F*_*(2, 164)*_ = 89.80, *p* < 0.001, *η*^*2*^_*(part)*_ = 0.52 *BPD* vs. *CC:* 7.06 ± 0.88^*^, 95% CI [− 4.97, 9.16] *BPD* vs. *HC:* 11.58 ± 0.87^*^, 95% CI [9.52, 13.63] *CC* vs. *HC:* 4.51 ± 0.87^*^, 95% CI [2.45, 6.58]
CTQ Emotional Neglect	17.45 ± 5.86	11.70 ± 4.91	6.95 ± 2.37	*F*_*(2, 164)*_ = 74.14, *p* < 0.001, *η*^*2*^_*(part)*_ = 0.48 *BPD* vs. *CC:* 5.75 ± 0.88^*^, 95% CI [− 3.67, 7.83] *BPD* vs. *HC:* 10.51 ± 0.86^*^, 95% CI [8.46, 12.55] *CC* vs. *HC:* 4.76 ± 0.87^*^, 95% CI [2.70, 6.81]
CTQ Physical Abuse	9.85 ± 5.09	6.59 ± 2.87	5.16 ± 0.62	*F*_*(2, 164)*_ = 28.72, *p* < 0.001, *η*^*2*^_*(part)*_ = 0.26 *BPD* vs. *CC:* 3.26 ± 0.64^*^, 95% CI [1.74, 4.79] *BPD* vs. *HC:* 4.70 ± 0.63^*^, 95% CI [3.20, 6.20] *CC* vs. *HC:* 1.44 ± 0.64, 95% CI [− 0.07, 2.94]
CTQ Physical Neglect	10.49 ± 3.88	6.98 ± 2.57	6.29 ± 1.75	*F*_*(2, 164)*_ = 34.63, *p* < 0.001, *η*^*2*^_*(part)*_ = 0.30 *BPD* vs. *CC:* 3.51 ± 0.55^*^, 95% CI [2.22, 4.80] *BPD* vs. *HC:* 4.20 ± 0.54^*^, 95% CI [2.93, 5.47] *CC* vs. *HC:* 0.69 ± 0.54, 95% CI [− 0.59, 1.97]
CTQ Sexual Abuse	11.55 ± 6.62	6.78 ± 4.03	5.33 ± 1.23	*F*_*(2, 164)*_ = 29.24, *p* < 0.001, *η*^*2*^_*(part)*_ = 0.26 *BPD* vs. *CC:* 4.77 ± 0.86^*^, 95% CI [2.73, 6.81] *BPD* vs. *HC:* 6.22 ± 0.85^*^, 95% CI [4.22, 8.22] *CC* vs. *HC:* 1.45 ± 0.85, 95% CI [− 0.56, 3.46]

Table shows means ± standard deviations of scores and results of the multivariate analysis of variance, with post-hoc Tuckey tests

*BPD* Borderline Personality Disorder (patient group), *CC* Clinical Control group consisting by ADHD and SUD, *CTQ* Childhood Trauma Questionnaire, *DERS* Difficulties in Emotion Regulation Scale, *HC* Healthy control group, *UPPS* UPPS Impulsive Behaviour Scale

*p < 0.05, **p < 0.01, ***p < 0.001

### Multiple regression analyses (total effects)

#### Childhood maltreatment severity and impulsivity (path c)

The overall model was significant (*F*_(3,168)_ = 24.14, *p* < .0001, *R*^*2*^ = .295, *R*^*2*^_(adj)_ = .283, CI: [1.903, 4.209]), with childhood maltreatment severity being a significant predictor for UPPS scores (*B* = 0.027, *SE* = 0.004, *t*_(173)_ = 7.56, *p* < .0001, CI: [0.020, 0.034]), while controlling for age (*B* = − 0.004, *SE* = 0.011, *t*_(173)_ = 0.33, *p* = .742, CI: [− 0.026, 0.018]) and education (*B* = − 0.246, *SE* = 0.152, *t*_(173)_ = 2.33, *p* = .021, CI: [− 0.455, − 0.037]). Among the subscales, emotional maltreatment (*B* = 0.065, *SE* = 0.010, *t*_(168)_ = 6.40, *p* < .0001, CI: [0.045, 0.086]) and physical maltreatment (*B* = − 0.043, *SE* = 0.019, *t*_(168)_ = 2.26, *p* = .025, CI: [− 0.081, − 0.010]) were significant predictors, while sexual abuse had no unique significant effect (*B* = 0.020, *SE* = 0.017, *t*_(168)_ = 1.18, *p* = .240, CI: [− 0.014, 0.054]).

#### Childhood maltreatment severity and difficulties in emotion regulation (path a)

The overall model was significant (*F*_(3,172)_ = 4.26, *p* = .006, *R*^*2*^ = .069, *R*^*2*^_(adj)_ = .053, CI: [89.067, 137.190]), with childhood maltreatment severity being a significant positive predictor for DERS scores (*B* = 0. 221, *SE* = 0.084, *t*_(172)_ = 2.52, *p* = .013, CI: [0.046, 0.377]), while controlling for age (*B* = 0.236, *SE* = 0.262, *t*_(172)_ = 0.262, *p* = .369, CI: [− 0.754, 0.281]) and education (*B* = − 5.039, *SE* = 02. 52, *t*_(172)_ = 2.00, *p* = .047, CI: [− 10.013, − 0.065]). Emotional maltreatment was again a unique significant predictor (*B* = 1.384, *SE* = 0.414, *t*_(172)_ = 3.35, *p* = .001, CI: [0.568, 2.201]. Neither physical maltreatment (*B* = − 0.904, *SE* = 0.778, *t*_(172)_ = 1.16, *p* = .247, CI: [− 2.440, 0.633]) nor sexual abuse (*B* = − 0.248, *SE* = 0.410, *t*_(172)_ = 0.61, *p* = .547, CI: [− 1.057, 0.562]) were significant predictors.

#### Difficulties in emotion regulation and impulsivity (path b)

The underlying association between the statistical mediator variable (DERS) and outcome (impulsivity, UPPS) could also be established (*F*_(3,173)_ = 18.27, *p* < .001, *R*^*2*^ = .241, *R*^*2*^_(adj)_ = .227, CI: [1.833, 4.130]). More difficulties in emotion regulation predicted more impulsivity (*B* = 0.021, *SE* = 0.003, *t*_(173)_ = 2.25, *p* < .0001, CI: [0.015, 0.028]), when controlling for age (*B* = 0.010, *SE* = 0.011, *t*_(173)_ = 0.86, *p* = .392, CI: [− 013, 0.32]), and education (*B* = − 0.250, *SE* = 0.111, *t*_(173)_ = 2.25, *p* = .025, CI: [− 0.468, − 0.031]). Thus, results suggest that a statistical mediation effect may occur.

### Conditional mediation analysis

The overall regression model was significant (*F*_(7,164)_ = 179.29, *p* < .0001, *R*^2^ = .884), suggesting that approximately 88% of the variance in self-reported impulsivity (UPPS mean scores) was explained by all predictors in the model. Specifically, higher levels of childhood maltreatment severity (*B* = 0.035, *SE* = 0.004, *t* = 8.26, *p < .*0001; CI: [0.026, 0.044]) and more difficulties in emotion regulation (*B* = 0.010, *SE* = 0.003, *t* = 3.27, *p = .*001; CI: [0.004, 0.015]) predicted more impulsivity. Group, age, and education also had significant effects, with younger age and lower education being related to higher impulsivity (age: *B* = − 0.013, *SE* = 0.005, *t* = 2.67, *p = .*008; CI: [− 0.022, − 0.003]; education: *B* = − 0.091, *SE* = 0.045, *t* = 2.02, *p = .*045; CI: -[0.180, − 0.002]). The effect of group was also significant (*B* = 2.070, *SE* = 0.185, *t* = 11.16, *p < .*0001; CI: [1.70, 2.434]).

**Fig. 4:**
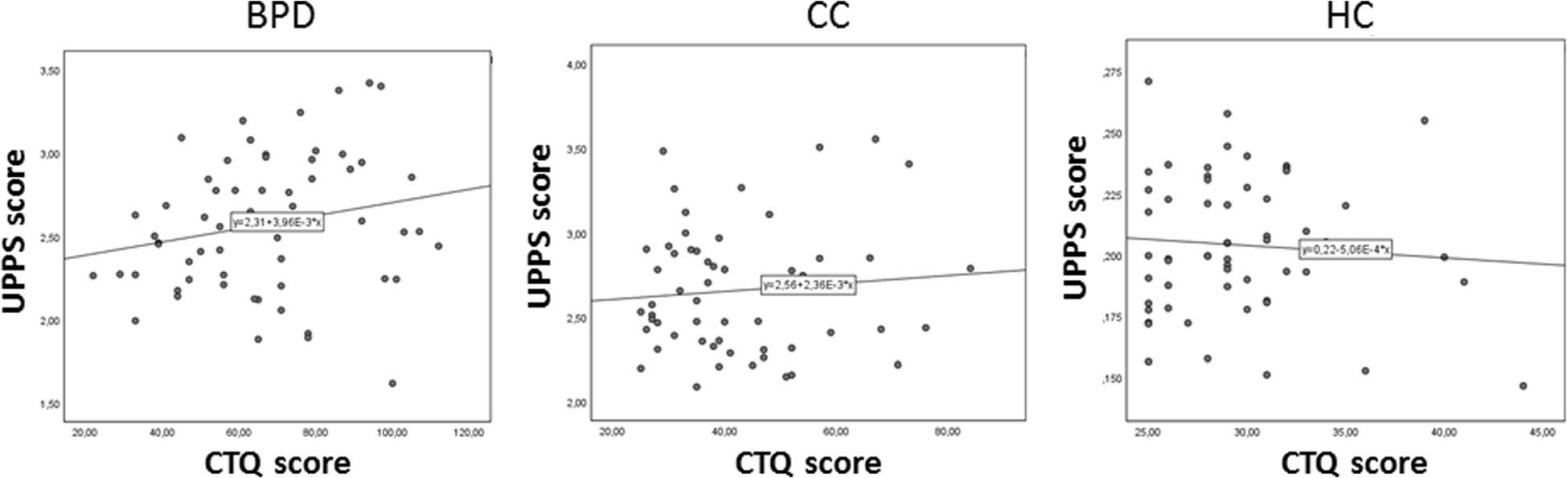
This scatterplot illustrates correlations between scores on the Childhood Trauma Questionnaire (CTQ) and scores on the UPPS Impulsive Behaviour Scale in patients with Borderline Personality Disorder (BPD), Clinical Controls (CC) and Healthy Controls (HC)

There was a significant interaction of childhood maltreatment severity and group on impulsivity (*B* = 0.017, *SE* = 0.004, *t* = 2.67, *p < .*0001; CI: [0.024, 0.010]). Within the three groups, childhood maltreatment severity positively predicted impulsivity in BPD (Rho = .232, *p* = .037, R^2^ = .05) but not in HC and ADHD (*p* > .05), see Fig. 4. The interaction between group and childhood trauma in predicting DERS was not significant (*B* = 0.019, *SE* = 0.188, *t* = 0.10, *p = .*917; CI: [− 0.392, 0.352]; CTQ: *B* = 0.154, *SE* = 0.212, *t* = 0.72, *p = .*471; CI: [− 0.266, 0.573]).

**Fig. 5:**
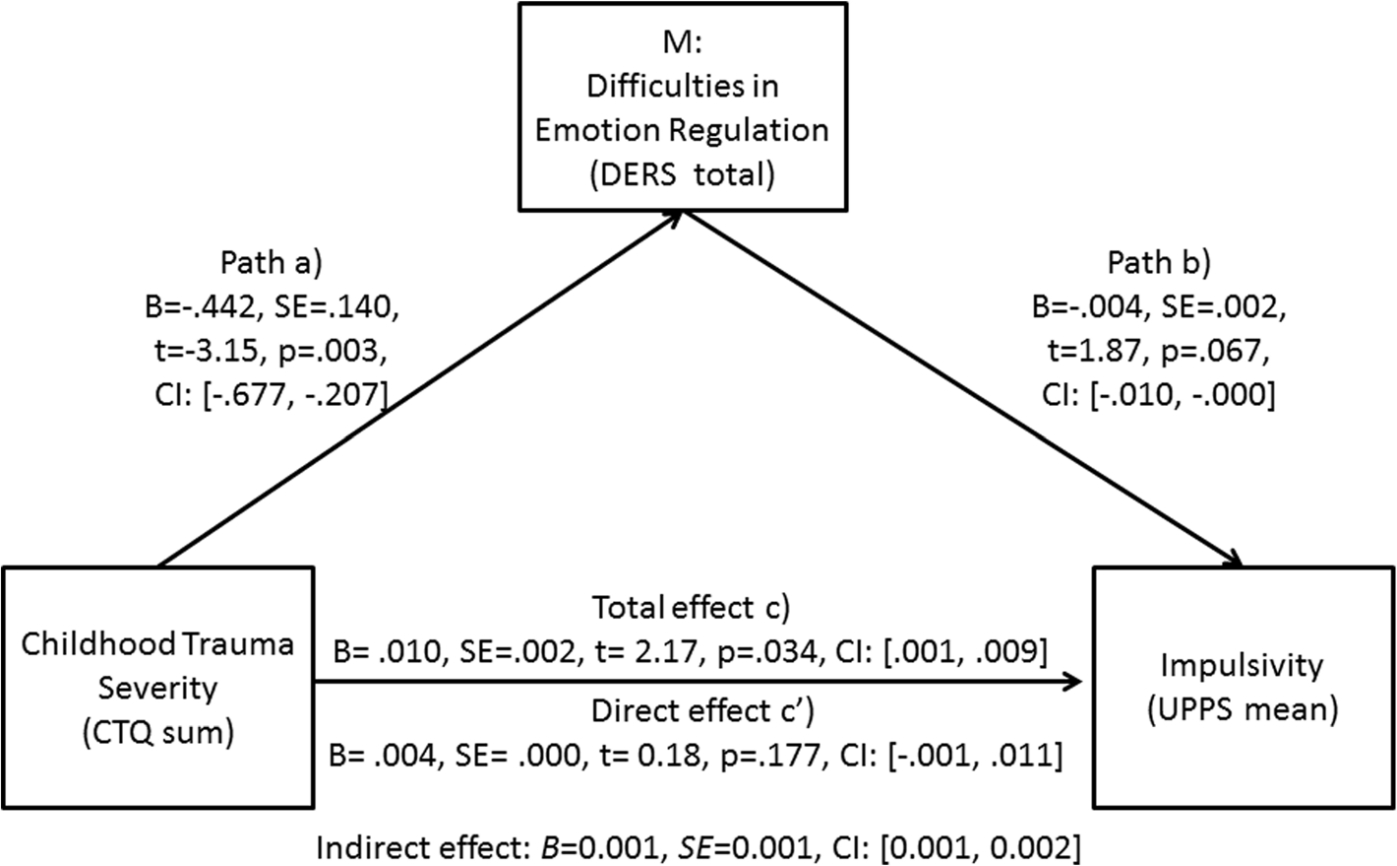
This Figure summarizes the effect of the mediation analysis in the group of Borderline Personality Disorder (BPD) patients

There was a significant interaction between DERS and group (*B* = − 0.005, *SE* = 0.002, *t* = 4.61, *p = .*032; CI: [− 0.010, − 0.0004]). Furthermore, there was a conditional effect of group regarding the effect of childhood maltreatment through difficulties in emotion regulation on impulsivity: Based on the bootstrapping confidence interval, difficulties in emotion regulation statistically mediated the effect of childhood trauma on impulsivity in the BPD group (*B* = 0.001, *SE* = 0.001, CI: [0.001, 0.002]) but not in the other groups (HC: *B* = 0.001, *SE* = 0.002, CI: [− 0.002, 0.006]; CC: *B* = 0.0001, *SE* = 0.001, CI: [− 0.004, 0.002]). As shown in Fig. 5, in the BPD group, childhood trauma had a significant effect on DERS (path a), which in turn significantly predicted impulsivity (path b). The total effect of childhood maltreatment on impulsivity was significant, while this link was not significant anymore, when controlling for DERS in the regression model.

Analyses for the four UPPS subscales separately revealed similar results. Analyses without the UPPS subscale Urgency revealed the same results (see Additional file 6).

## Discussion

This self-report study aimed at investigating the effect of childhood maltreatment severity on impulsivity and whether difficulties in emotion regulation statistically mediated this relationship in BPD compared to healthy controls and clinical controls. Higher childhood maltreatment severity, particularly emotional maltreatment, predicted more difficulties in emotion regulation and impulsivity across all groups. There was a significant interaction effect of childhood maltreatment and group in predicting impulsivity: The effect of childhood maltreatment severity on impulsivity was significantly more pronounced in BPD than in HC and ADHD. Moreover, a significant statistical mediation effect was found, depending on group: In the BPD group, the effect of childhood maltreatment on impulsivity in BPD was not significant anymore, when controlling for difficulties in emotion regulation.

The positive association between childhood maltreatment severity, difficulties in emotion regulation, and impulsivity is in line with our hypothesis and previous research [14–21, 32–38]. Childhood maltreatment can have devastating effects on the development of healthy and adaptive emotion regulation and self-control, e.g., the ability to tolerate intense negative emotions, considering the results of one’s actions, and to focus on goal-directed behaviour when in a negative state [22, 23].

Among the different types of childhood maltreatment, emotional maltreatment was the only significant predictor for both emotion regulation difficulties and impulsivity. Emotional maltreatment is considered a particularly chronic and detrimental form of abuse. This may involve humiliating or demeaning behaviour toward the child, psychological unavailability of caretakers (e.g., due to illness) and a failure to meet children’s basic emotional and psychological needs – often a consequence of the parent’s own unresolved childhood adversities [64–68]. A history of emotional maltreatment has been directly linked to alterations in emotional processing, including increased affect intensity and decreased distress tolerance [64–67]. Among different forms of child maltreatment, emotional abuse was the strongest predictor of emotion regulation difficulties later in life [66]. In particular, previous studies in BPD found that emotional maltreatment (emotional abuse and neglect) was the strongest predictor for malfunctioning emotion regulation strategies [68] and BPD symptom severity [20, 68], when controlling for other types of abuse [20, 68].

Further in line with previous studies, we found higher rates of childhood maltreatment in the BPD group compared to the other groups [14–21] as well as in the clinical control group compared to healthy controls [32–38]. The finding of higher childhood maltreatment rates in BPD is consistent with previous research; e.g., in the large-scale multicenter Collaborative Longitudinal Personality Disorders Study, higher rates of self-reported childhood abuse and neglect were found in individuals with BPD than in other personality disorders [15]. Moreover, among four groups of personality disorder (schizotypal, borderline, avoidant, and obsessive-compulsive), and a major depression comparison group, BPD participants reported the highest rate of traumatic exposure (particularly to sexual traumas, including childhood sexual abuse and being physically attacked), and youngest age of first traumatic event [21].

Interestingly, the effect of childhood maltreatment severity on impulsivity was significantly more pronounced in BPD than in the control groups. While a history of trauma is neither necessary nor sufficient for the etiology of BPD, childhood abuse, especially emotional and sexual abuse, was found to aggravate BPD symptomatology. This is in line with the biosocial theory by Linehan [27] and current conceptualizations of BPD highlighting the role of an invalidating or traumatic environment in the etiology of the disorder [2]. At the same time, childhood maltreatment may put individuals at higher risk for developing other psychopathologies, such as ADHD and SUD, which frequently co-occur with BPD [32–38].

In the present study, emotion regulation difficulties statistically mediated the relationship between childhood maltreatment severity and impulsivity in BPD, but not in the other groups. Since impulsive behaviour in BPD mainly occurs under emotional distress [9–12], it has been conceptualized as a consequence or facet of malfunctioning emotion regulation mechanisms [2, 3]. In previous research, stress-dependent increases in impulsivity were found in BPD but not in adults with Attention Deficit Hyperactivity Disorder (ADHD) [9, 11]. In line with this and in the context of other previous experimental and neurobiological research [9–13], our findings suggest that difficulties in emotion regulation may underlie self-perceived impulsive behaviour in BPD.

While the inclusion of well-characterized patient groups and the exclusion of mutual comorbidity between the groups (i.e., BPD, SUD/ADHD) is a clear strength of our study, this strict recruitment restricted our samples sizes, which may have limited the statistical power to detect effects, especially in smaller subsamples (ADHD, SUD patients). While we only included women, further research should also include male participants. Since the cross-sectional correlational design of our study does not allow causal conclusions, prospective and longitudinal studies with larger samples are required to gain more insights into causal relationships. In particular, to replicate the statistical mediation effect observed in our study and to identify a directional, potentially causal link, studies with experimental and/or repeated-measure data are needed, in which the independent variable precedes the dependent variable in time. This is of particular importance since mediation analyses with cross-sectional data can lead to an over-estimation of effects [69]. Likewise, the use of self-reports generally involves the risk of potential biases, such as social desirability, limited awareness and insight, different subjective interpretations of measured concepts, and/or a ‘coloring’ of reports by current mood [70]. Childhood maltreatment was assessed in a retrospective and subjective manner which is particularly prone to recall biases. It is possible that individuals with BPD may suffer from more traumatic re-experiencing, associated with more vivid negative memories, and may consequently recall childhood experiences more negatively or have a tendency to report more negative childhood adversities.

Previous research suggests that there self-reports and behavioural or psychophysiological measures of emotion regulation and impulsivity in BPD are only weakly or not at all correlated [29–31]. Therefore, future research should additionally use experimental tasks of emotion regulation (e.g., cognitive reappraisal task), impulsivity (e.g., Go/NoGo tasks, stop signal tasks, or delay discounting task) and emotional distress (e.g., experimental psychosocial stressor tasks) [9–13], preferably combining multiple measures (self-reports, behavioural tasks, psychophysiological measures such as heart rate or skin conductance, neuroimaging) at different assessments points.

Including a control group of healthy participants who had been exposed to severe childhood maltreatment without having developed a mental disorder would help corroborating associations between childhood maltreatment, emotion dysregulation, and impulsivity. In general, a full factorial design, with additional control groups for high versus low levels of childhood maltreatment in HC as well as BPD and CC would allow a better investigation of the impact of childhood adversities on impulsivity (and interactions with mental disorders such as BPD). Future research may take the duration and onset of childhood trauma into account to further extend our findings.

A remaining research question is to which degree different components of emotion regulation and multiple facets of impulsivity overlap or can be disentangled from each other. In a previous study, we showed that deficits in action withholding / response inhibition (Go/NoGo task) were influenced by acute experimental stress, while delay discounting was a more stable feature in BPD [11]. To identity common and distinct components of impulsivity in relation to emotion regulation in BPD, ADHD, and SUD, future research might employ network analyses, aimed at visualizing inter-relations (node strengths centrality) between factors in a pre-defined model. For instance, this method might help addressing the centrality of factors, such as childhood adversities, emotion dysregulation, and impulsivity, and their place in a network, i.e., how distinct and/or connected these factors are in predicting BPD severity [71].

Since positive urgency (acting impulsively while experiencing extreme positive affect) was not assessed in our study, future research should investigate this factor of impulsivity in more detail. Likewise, the DERS mainly focuses on negative feelings of emotional distress. Typical expressions of impulsivity in BPD, such as gambling, substance abuse, promiscuity, or risky sexual activities, may not only serve reducing negative feelings but also increasing positive feelings (e.g., joy, excitement, belonging), which can have devastating consequences on physical/mental health and across different life domains (work, relationships, etc.).

More research is needed to replicate our novel findings and to gain deeper insight into other factors (e.g., positive emotions) that may contribute to impulsivity in BPD.

## Conclusion

In conclusion, our findings point to a significant association between childhood maltreatment severity, difficulties in emotion regulation, and impulsivity in BPD. Emotion dysregulation was found to underlie self-perceived impulsive behaviour in BPD. Strengthening emotion regulation strategies, especially in interpersonal contexts, is a main focus of evidence-based BPD treatments, such as Dialectical Behavioural Therapy [27], Mentalization-Based Therapy [72], Transference-focused psychotherapy [73], and Schema Therapy [74]. In combination with emotion regulation training, addressing the consequences of childhood adversities (e.g., using psychoeducation to highlight associations with stress tolerance and impulsivity) and integrating traumatic experiences into autobiographical memory (e.g., exposure-based treatment in combination with skills training and stabilizing interventions) might help to reduce impulsive behaviour, such as self-harm and suicidal attempts, in BPD.

## Additional files

Additional file 1: **Table S1*.*** Demographic characteristics of patients with Borderline Personality Disorder (BPD), subgroups of patients with Attention Deficit Hyperactivity Disorder (ADHD) and Substance Use Disorder (SUD) and Healthy Controls (HC). (DOCX 19 kb)

Additional file 2: **Table S2.** Descriptive values and results of the MANOVA for the Childhood Trauma Questionnaire (CTQ) in patients with Borderline Personality Disorder (BPD), subgroups of patients with Attention Deficit Hyperactivity Disorder (ADHD) and Substance Use Disorder (SUD) and Healthy Controls (HC). (DOCX 21 kb)

Additional file 3: **Table S3.** Descriptive values and results of the MANOVA for the UPPS Impulsive Behaviour Scale in patients with Borderline Personality Disorder (BPD), subgroups of patients with Attention Deficit Hyperactivity Disorder (ADHD) and Substance Use Disorder (SUD) and Healthy Controls (HC). (DOCX 20 kb)

Additional file 4: **Table S4.** Descriptive values and results of the MANOVA for the Difficulties in Emotion Regulation Scale (DERS) in patients with Borderline Personality Disorder (BPD), subgroups of patients with Attention Deficit Hyperactivity Disorder (ADHD) and Substance Use Disorder (SUD) and Healthy Controls (HC). (DOCX 22 kb)

Additional file 5: **Table S5.** Correlations between Scores on the scales. (DOCX 24 kb)

Additional file 6: Conditional process analysis without the subscale Urgency. (DOCX 13 kb)

## Footnotes

1 We decided to not split the CC group into subgroups of patients with ADHD and SUD, because these subsamples were too small to perform a meaningful group comparison and regression analysis. ADHD and SUD did not differ significantly in age (Additional file 1: Table S1) and ACE (Additional file 2: Table S2) and together showed similarly high levels of impulsivity and emotion regulation difficulties as the BPD group.

2 In Process model 59, there is no option for multi-categorical variables. Therefore, the analysis was re-run with changed group labels, which ensured that results were robust.

3 In Process model 59, there is no option for a total effect model, therefore separate MRAs were performed.
