# Supplementary material for: Self-reported impulsivity in women with borderline personality disorder: the role of childhood maltreatment severity and emotion regulation difficulties
Source: Borderline Personal Disord Emot Dysregul. 2019 Mar 5;6:6. doi: 10.1186/s40479-019-0101-8 (PMC6399941; doi:10.1186/s40479-019-0101-8)
Supplement: Supplementary file 1 — Table S1. Demographic characteristics of patients with Borderline Personality Disorder (BPD), subgroups of patients with Attention Deficit Hyperactivity Disorder (ADHD) and Substance Use Disorder (SUD) and Healthy Controls (HC). (DOCX 19 kb) [file 40479_2019_101_MOESM1_ESM.docx]

Table S1

Demographic characteristics of patients with Borderline Personality Disorder (BPD), subgroups of patients with Attention Deficit Hyperactivity Disorder (ADHD) and Substance Use Disorder (SUD) and Healthy Controls (HC)

| **Clinical Controls** | | | | | **Results of**  **t-test / Chi² test** |
| --- | --- | --- | --- | --- | --- |
| **Variable** | **BPD**  (n=61) | **ADHD**  (n=28) | **SUD**  (n=28) | **HC**  (n=60) |  |
| Age | 27.28 *±* 6.84 | 30.11 *±* 6.96 | 31.14 *±* 8.35 | 27.60 *±* 6.84 | *F _(_*_3, 173)_= 2.86 , *p =* 0.038, *η^2^_(part)_* = 0.047  *BPD vs. SUD:* -3.86 *±* 1.57, 95% CI [-7.94, 0.21]  *BPD vs. ADHD:* -2.83 *±* 1.57, 95% CI [-6.91, 1.25]  *BPD vs. HC:* -0.32 *±* 1.25, 95% CI [-3.57, 2.93]  *ADHD vs. HC:* 2.51 *±* 1.58, 95% CI [-1.58, 6.60]  *SUD vs. HC:* 3.54 ± 1.58, 95% CI: [-0.55, 7.63]  *ADHD vs. SUD:* -1.04 *±* 1.84, 95% CI [-5.81, 3.74] |
| Education |  |  |  |  |  |
| No education | 1 | 0 | 4 | 0 |  |
| 9 years | 7 | 0 | 3 | 1 | Chi²(9) = 48.61, *p* < 0.001 |
| 10 years | 25 | 0 | 10 | 14 |  |
| 12-13 years | 28 | 28 | 11 | 45 |  |

Note: Table shows means ± standard deviations of age and frequency of education.
