# Supplementary material for: Self-reported impulsivity in women with borderline personality disorder: the role of childhood maltreatment severity and emotion regulation difficulties
Source: Borderline Personal Disord Emot Dysregul. 2019 Mar 5;6:6. doi: 10.1186/s40479-019-0101-8 (PMC6399941; doi:10.1186/s40479-019-0101-8)
Supplement: Supplementary file 2 — Table S2. Descriptive values and results of the MANOVA for the Childhood Trauma Questionnaire (CTQ) in patients with Borderline Personality Disorder (BPD), subgroups of patients with Attention Deficit Hyperactivity Disorder (ADHD) and Substance Use Disorder (SUD) and Healthy Controls (HC). (DOCX 21 kb) [file 40479_2019_101_MOESM2_ESM.docx]

Table S2

Descriptive values and results of the MANOVA for the Childhood Trauma Questionnaire (CTQ) in patients with Borderline Personality Disorder (BPD), subgroups of patients with Attention Deficit Hyperactivity Disorder (ADHD) and Substance Use Disorder (SUD) and Healthy Controls (HC)

|  |  | **Clinical Controls** | |  |  |
| --- | --- | --- | --- | --- | --- |
| **Variable** | **BPD**  (n=61) | **ADHD**  (n=28) | **SUD**  (n=28) | **HC**  (n=60) | **Group statistics** |
| **Childhood Trauma Questionnaire**  Sum score | 67.67 ± 21.60 | 44.96 ± 15.87 | 39.67 ± 13.14 | 30.77 ± 7.34 | *F_(3, 168)_* = 57.54, *p* < 0.001, *η^2^_(part)_* = 0.51  *BPD vs. SUD:* 28.00 ± 3.63***, 95% CI [18.58, 37.42]  *BPD vs. ADHD:* 22.70 ± 3.59***, 95% CI [13.39, 32.01]  *BPD vs. HC:* 36.90 ± 2.87***, 95% CI [29.44, 44.36]  *ADHD vs. HC:* 14.20 ± 3.56**, 95% CI [4.97, 23.43]  *ADHD vs. SUD:* 5.30 ± 4.19, 95% CI [-5.58, 16.18]  *SUD vs. HC:* 8.90 ± 3.60, 95% CI [-0.45, 18.25] |
| CTQ Emotional Abuse | 17.70 ± 6.10 | 11.25 ± 5.73 | 9.59 ± 4.21 | 6.25 ± 2.06 | *F_(3, 168)_* = 60.00, *p* < 0.001, *η^2^_(part)_* = 0.52  *BPD vs. SUD:* 8.11 ± 1.09***, 95% CI [5.27, 10.95]  *BPD vs. ADHD:* 6.45 ± 1.08***, 95% CI [3.65, 9.25]  *BPD vs. HC:* 11.45 ± 0.87***, 95% CI [9.21, 13.70]  *ADHD vs. HC:* 5.00 ± 1.07***, 95% CI [2.22, 7.78]  *ADHD vs. SUD:* 1.66 ± 1.26, 95% CI [-1.62, 4.93]  *SUD vs. HC:* 3.34 ± 1.08*, 95% CI [0.53, 6.16] |
| CTQ Emotional Neglect | 17.63 ± 5.85 | 12.71 ± 4.85 | 10.56 ± 4.74 | 7.47 ± 3.67 | *F_(3, 168)_* = 44.28, *p* < 0.001, *η^2^_(part)_* = 0.44  *BPD vs. SUD:* 7.08 ± 1.13***, 95% CI [4.14, 10.01]  *BPD vs. ADHD:* 4.92 ± 1.12***, 95% CI [2.02, 7.81]  *BPD vs. HC:* 10.17 ± 0.89***, 95% CI [7.84, 12.49]  *ADHD vs. HC:* 5.25 ± 1.11***, 95% CI [2.38, 8.12]  *ADHD vs. SUD:* 2.16 ± 1.30, 95% CI [-1.23, 5.54]  *SUD vs. HC:* 3.09 ± 1.12*, 95% CI [0.18, 6.00`] |
| CTQ Physical Abuse | 10.04 ± 5.21 | 6.64 ± 3.12 | 6.48 ± 2.59 | 5.38 ± 1.66 | *F_(3, 168)_* = 17.87, *p* < 0.001, *η^2^_(part)_* = 0.24  *BPD vs. SUD:* 3.55 ± 0.83***, 95% CI [1.40, 5.71]  *BPD vs. ADHD:* 3.39 ± 0.82***, 95% CI [1.26, 5.52]  *BPD vs. HC:*  4.65 ± 0.66***, 95% CI [2.95, 6.36]  *ADHD vs. HC:* 1.26 ± 0.81, 95% CI [-0.85, 3.37]  *ADHD vs. SUD:* 0.16 ± 0.96, 95% CI [-2.33, 2.65]  *SUD vs. HC:* 1.10 ± 0.82, 95% CI [-1.04, 3.24] |
| CTQ Physical Neglect | 10.56 ± 3.89 | 7.79 ± 3.10 | 6.11 ± 1.42 | 6.30 ± 1.74 | *F_(3, 168)_* = 26.99, *p* < 0.001, *η^2^_(part)_* = 0.33  *BPD vs. SUD:* 4.45 ± 0.66***, 95% CI [2.74, 6.16]  *BPD vs. ADHD:* 2.78 ± 0.65***, 95% CI [1.09, 4.46]  *BPD vs. HC:* 4.26 ± 0.52***, 95% CI [2.91, 5.61]  *ADHD vs. HC:* 1.49 ± 0.65, 95% CI [-0.19, 3.16]  *ADHD vs. SUD:* 1.67 ± 0.76, 95% CI [-0.30, 3.65]  *SUD vs. HC:* -0.19 ± 0.65, 95% CI [-1.88, 1.51] |
| CTQ Sexual Abuse | 11.74 ± 6.75 | 6.57 ± 3.86 | 6.93 ± 4.21 | 5.37 ± 1.26 | *F_(3, 168)_* = 20.73, *p* < 0.001, *η^2^_(part)_* = 0.27  *BPD vs. SUD:* 4.81 ± 1.07***, 95% CI [2.04, 7.58]  *BPD vs. ADHD:* 5.17 ± 1.05***, 95% CI [2.43, 7.90]  *BPD vs. HC:* 6.37 ± 0.85***, 95% CI [4.18, 8.56]  *ADHD vs. HC:* 1.20 ± 1.05, 95% CI [-1.51, 3.92]  *ADHD vs. SUD:* -0.35 ± 1.23, 95% CI [-3.55, 2.84]  *SUD vs. HC:* 1.56 ± 1.06, 95% CI [-1.19, 4.31] |

*Note*. Table shows means ± standard deviations of scores and results of the multivariate analysis of variance, with post-hoc Tuckey tests; BPD = Borderline Personality Disorder (patient group); CTQ = Childhood Trauma Questionnaire. **p* < 0.05, ***p* < 0.01, ****p* < 0.001
