# Supplementary material for: Self-reported impulsivity in women with borderline personality disorder: the role of childhood maltreatment severity and emotion regulation difficulties
Source: Borderline Personal Disord Emot Dysregul. 2019 Mar 5;6:6. doi: 10.1186/s40479-019-0101-8 (PMC6399941; doi:10.1186/s40479-019-0101-8)
Supplement: Supplementary file 3 — Table S3. Descriptive values and results of the MANOVA for the UPPS Impulsive Behaviour Scale in patients with Borderline Personality Disorder (BPD), subgroups of patients with Attention Deficit Hyperactivity Disorder (ADHD) and Substance Use Disorder (SUD) and Healthy Controls (HC). (DOCX 20 kb) [file 40479_2019_101_MOESM3_ESM.docx]

Table S3

Descriptive values and results of the MANOVA for the UPPS Impulsive Behaviour Scale in patients with Borderline Personality Disorder (BPD), subgroups of patients with Attention Deficit Hyperactivity Disorder (ADHD) and Substance Use Disorder (SUD) and Healthy Controls (HC)

|  |  | **Clinical Controls** | |  |  |
| --- | --- | --- | --- | --- | --- |
| **Variable** | **BPD**  (n=61) | **ADHD**  (n=28) | **SUD**  (n=28) | **HC**  (n=60) | **Group statistics** |
| **UPPS Scale**  Negative Urgency | 2.67 ± 0.85 | 3.04 ± 0.50 | 2.63 ± 0.51 | 0.22 ± 0.06 | *F_(3, 174)_* = 258.73, *p* < 0.001, *η^2^_(part)_* = 0.82  *BPD vs. SUD:* 0.04 ± 0.13, 95% CI [-0.30, 0.38]  *BPD vs. ADHD:* -0.37 ± 0.13*, 95% CI [-0.71, -0.03]  *BPD vs. HC:* 2.46 ± 0.10***, 95% CI [2.19, 2.73]  *ADHD vs. HC:* 2.82 ± 0.13***, 95% CI [2.48, 3.17]  *ADHD vs. SUD:* 0.41 ± 0.15*, 95% CI [0.01, 0.81]  *SUD vs. HC:* 2.42 ± 0.13***, 95% CI [2.07, 2.76] |
| Premeditation | 2.23 ± 0.49 | 2.75 ± 0.53 | 2.18 ± 0.42 | 0.178 ± 0.03 | *F_(3, 174)_* = 408.89, *p* < 0.001, *η^2^_(part)_* = 0.88  *BPD vs. SUD:* 0.05 ± 0.09, 95% CI [-0.18, 0.29]  *BPD vs. ADHD:* -0.52 ± 0.09***, 95% CI [-0.75, -0.28]  *BPD vs. HC:* 2.05 ± 0.07***, 95% CI [1.87, 2.24]  *ADHD vs. HC:* 2.57 ± 0.09***, 95% CI [2.33, 2.80]  *ADHD vs. SUD:* 0.57 ± 0.11***, 95% CI [0.30, 0.85]  *SUD vs. HC:* 2.00 ± 0.09***, 95% CI [1.76, 2.23] |
| Perseverance | 2.39 ± 0.61 | 2.88 ± 0.44 | 2.29 ± 0.20 | 0.26 ± 0.05 | *F_(3, 174)_* = 400.05, *p* < 0.001, *η^2^_(part)_* = 0.87  *BPD vs. SUD:* 0.10 ± 0.09, 95% CI [-0.14, 0.34]  *BPD vs. ADHD:* -0.49 ± 0.09***, 95% CI [-0.73, -0.25]  *BPD vs. HC:* 2.13 ± 0.07***, 95% CI [1.94, 2.33]  *ADHD vs. HC:* 2.62 ± 0.09***, 95% CI [2.38, 2.87]  *ADHD vs. SUD:* 0.59 ± 0.11***, 95% CI [0.31, 0.88]  *SUD vs. HC:* 2.03 ± 0.09***, 95% CI [1.79, 2.27] |
| Sensation Seeking | 2.30 ± 0.82 | 2.94 ± 0.80 | 2.52 ± 0.45 | 0.17 ± 0.04 | *F_(3, 174)_* = 269.53, *p* < 0.001, *η^2^_(part)_* = 0.82  *BPD vs. SUD:* 0.48 ± 0.14**, 95% CI [0.12, 0.84]  *BPD vs. ADHD:* 0.06 ± 0.14, 95% CI [-0.30, 0.41]  *BPD vs. HC:* 2.83 ± 0.11***, 95% CI [2.55, 3.12]  *ADHD vs. HC:* 2.78 ± 0.14***, 95% CI [2.42, 3.14]  *ADHD vs. SUD:* 0.42 ± 0.16*, 95% CI [0.002, 0.84]  *SUD vs. HC:* 2.36 ± 0.14***, 95% CI [2.00, 2.72] |

*Note*. Table shows means ± standard deviations of scores and results of the multivariate analysis of variance, with post-hoc Tuckey tests; BPD = Borderline Personality Disorder (patient group); HC = Healthy control group; UPPS = UPPS Impulsive Behaviour Scale. **p* < 0.05, ***p* < 0.01, ****p* < 0.001
