# Supplementary material for: Self-reported impulsivity in women with borderline personality disorder: the role of childhood maltreatment severity and emotion regulation difficulties
Source: Borderline Personal Disord Emot Dysregul. 2019 Mar 5;6:6. doi: 10.1186/s40479-019-0101-8 (PMC6399941; doi:10.1186/s40479-019-0101-8)
Supplement: Supplementary file 4 — Table S4. Descriptive values and results of the MANOVA for the Difficulties in Emotion Regulation Scale (DERS) in patients with Borderline Personality Disorder (BPD), subgroups of patients with Attention Deficit Hyperactivity Disorder (ADHD) and Substance Use Disorder (SUD) and Healthy Controls (HC). (DOCX 22 kb) [file 40479_2019_101_MOESM4_ESM.docx]

Table S4

Descriptive values and results of the MANOVA for the Difficulties in Emotion Regulation Scale (DERS) in patients with Borderline Personality Disorder (BPD), subgroups of patients with Attention Deficit Hyperactivity Disorder (ADHD) and Substance Use Disorder (SUD) and Healthy Controls (HC)

|  |  | **Clinical Controls** | |  |  |
| --- | --- | --- | --- | --- | --- |
| **Variable** | **BPD**  (n=58) | **ADHD**  (n=27) | **SUD**  (n=28) | **HC**  (n=60) | **Group statistics** |
| DERS  total score | 97.52 ± 13.32 | 104.37 ± 18.90 | 79.86 ± 20.50 | 69.47 ± 13.32 | *F_(3, 169)_* = 29.27, *p* < 0.001, *η^2^_(part)_* = 0.34  *BPD vs. SUD:* 17.66 ± 4.55**, 95% CI [5.86, 29.46]  *BPD vs. ADHD:* -6.85 ± 4.60, 95% CI [-18.80, 5.09]  *BPD vs. HC:* 28.05 ± 3.64***, 95% CI [18.61, 37.49]  *ADHD vs. HC:* 34.90 ± 4.58***, 95% CI [23.02, 46.78]  *ADHD vs. SUD:* 24.51 ± 5.33***, 95% CI [10.69, 38.34]  *SUD vs. HC:* 10.39 ± 4.52, 95% CI [-1.34, 22.12] |
| DERS  Clarity | 12.55 ± 4.77 | 15.78 ± 4.62 | 9.86 ± 2.90 | 8.08 ± 2.54 | *F_(3, 169)_* = 29.83, *p* < 0.001, *η^2^_(part)_* = 0.35  *BPD vs. SUD:* 2.69 ± 0.88*, 95% CI [0.42, 4.97]  *BPD vs. ADHD:* -3.23 ± 0.89**, 95% CI [-5.53, -0.92]  *BPD vs. HC:* 4.47 ± 0.70***, 95% CI [2.65, 6.29]  *ADHD vs. HC:* 7.69 ± 0.88***, 95% CI [5.40, 9.99]  *ADHD vs. SUD:* 5.92 ± 1.12***, 95% CI [-13.10, -7.30]  *SUD vs. HC:* 2.36 ± 0.14**, 95% CI [2.00, 2.72] |
| DERS Regulation strategies | 22.03 ± 7.92 | 25.33 ± 5.87 | 17.11 ± 5.79 | 11.83 ± 3.76 | *F_(3, 169)_* = 42.56, *p* < 0.001, *η^2^_(part)_* = 0.43  *BPD vs. SUD:* 4.93 ± 1.40**, 95% CI [1.31, 8.55]  *BPD vs. ADHD:* -3.30 ± 1.41, 95% CI [-6.96, 0.37]  *BPD vs. HC:* 10.20 ± 1.12***, 95% CI [7.30, 13.10]  *ADHD vs. HC:* 13.50 ± 1.40***, 95% CI [9.85, 17.15]  *ADHD vs. SUD:* 8.23 ± 1.64***, 95% CI [3.98, 12.47]  *SUD vs. HC:* 5.27 ± 1.39**, 95% CI [1.67, 8.87] |
| DERS Awareness | 16.14 ± 4.90 | 16.26 ± 2.96 | 14.79 ± 4.03 | 16.88 ± 3.60 | *F_(3, 169)_* = 1.70, *p* = 0.17, *η^2^_(part)_* = 0.03  *BPD vs. SUD:* 1.35 ± 0.94, 95% CI [-1.08, 3.78]  *BPD vs. ADHD:* -0.12 ± 0.95, 95% CI [-2.58, 2.34]  *BPD vs. HC:* -0.75 ± 0.75, 95% CI [-2.69, 1.20]  *ADHD vs. HC:* -0.62 ± 0.94, 95% CI [-3.07, 1.82]  *ADHD vs. SUD:* 1.47 ± 1.10, 95% CI [-1.38, 4.32]  *SUD vs. HC:* -2.10 ± 0.93, 95% CI [-4.52, 0.32] |
| DERS  Control | 18.22 ± 5.34 | 17.63 ± 4.47 | 11.15 ± 3.88 | 11.03 ± 2.78 | *F_(3, 169)_* = 39.41, *p* < 0.001, *η^2^_(part)_* = 0.41  *BPD vs. SUD:* 7.08 ± 0.97***, 95% CI [4.56, 9.60]  *BPD vs. ADHD:* 0.59 ± 0.98, 95% CI [-1.96, 3.15]  *BPD vs. HC:* 7.19 ± 0.78***, 95% CI [5.17, 9.21]  *ADHD vs. HC:* 6.60 ± 0.98***, 95% CI [4.06, 9.13]  *ADHD vs. SUD:* 6.49 ± 1.14***, 95% CI [3.53, 9.44]  *SUD vs. HC:* 0.11 ± 0.97, 95% CI [-2.40, 2.62] |
| DERS  Goals | 12.07 ± 5.63 | 10.59 ± 3.53 | 14.71 ± 5.22 | 11.33 ± 4.26 | *F_(3, 169)_* = 4.10, *p* = 0.008, *η^2^_(part)_* = 0.07  *BPD vs. SUD:* -2.65 ± 1.11, 95% CI [-5.53, 0.24]  *BPD vs. ADHD:* 1.48 ± 1.12, 95% CI [-1.44, 4.39]  *BPD vs. HC:* 0.74 ± 0.89, 95% CI [-1.57, 3.04]  *ADHD vs. HC:* -0.74 ± 1.12, 95% CI [-3.64, 2.16]  *ADHD vs. SUD:* -4.12 ± 1.30*, 95% CI [-7.50, -0.74]  *SUD vs. HC:* 3.38 ± 1.10*, 95% CI [0.51, 6.25] |
| DERS Acceptance | 15.50 ± 6.64 | 18.78 ± 6.42 | 12.25 ± 4.81 | 10.30 ± 3.39 | *F_(3, 169)_* = 21.75, *p* < 0.001, *η^2^_(part)_* = 0.28  *BPD vs. SUD:* 4.25 ± 1.24**, 95% CI [1.04, 7.46]  *BPD vs. ADHD:* -2.28 ± 1.25, 95% CI [-5.53, 0.97]  *BPD vs. HC:* 6.20 ± 0.99***, 95% CI [3.63, 8.78]  *ADHD vs. HC:* 8.48 ± 1.25***, 95% CI [5.24, 11.71]  *ADHD vs. SUD:* 6.53 ± 1.45***, 95% CI [2.77, 10.29]  *SUD vs. HC:* 1.95 ± 1.23, 95% CI [-1.24, 5.14] |

*Note*. Table shows means ± standard deviations of scores and results of the multivariate analysis of variance, with post-hoc Tuckey tests; BPD = Borderline Personality Disorder (patient group); DERS = Difficulties in Emotion Regulation Scale; HC = Healthy control group. **p* < 0.05, ***p* < 0.01, ****p* < 0.001
