# Supplementary material for: Self-reported impulsivity in women with borderline personality disorder: the role of childhood maltreatment severity and emotion regulation difficulties
Source: Borderline Personal Disord Emot Dysregul. 2019 Mar 5;6:6. doi: 10.1186/s40479-019-0101-8 (PMC6399941; doi:10.1186/s40479-019-0101-8)
Supplement: Supplementary file 5 — Table S5. Correlations between Scores on the scales. (DOCX 24 kb) [file 40479_2019_101_MOESM5_ESM.docx]

Table S5.

*Correlations Between Scores on the scales*

| Variables | 1 | 2 | 3 | 4 | 5 | 6 | 7 | 8 | 9 | 10 | 11 | 12 | 13 | 14 | 15 |
| --- | --- | --- | --- | --- | --- | --- | --- | --- | --- | --- | --- | --- | --- | --- | --- |
| 1. UPPS Urgency | - | 0.539*** | 0.543*** | 0.670*** | 0.319*** | 0.338*** | 0.221** | 0.078 | 0.006 | 0.34*** | 0.626*** | 0.482*** | 0.388*** | 0.298*** | 0.298*** |
| 1. UPPS Premeditation | 0.539*** | - | 0.820*** | 0.694*** | 0.236** | 0.323*** | 0.358*** | 0.079 | -0.121 | 0.36*** | 0.583*** | 0.489*** | 0.410*** | 0.438*** | 0.408*** |
| 1. UPPS Perseverance | 0.543*** | 0.820*** | - | 0.640*** | 0.253** | 0.355*** | 0.296*** | 0.153 | -0.131 | 0.35*** | 0.525*** | 0.482*** | 0.333*** | 0.389*** | 0.407*** |
| 1. UPPS Sensation Seeking | 0.670*** | 0.694*** | 0.640*** | - | 0.202* | 0.281** | 0.245** | -0.035 | -0.123 | 0.45*** | 0.639*** | 0.517*** | 0.411*** | 0.492*** | 0.472*** |
| 1. DERS Clarity | 0.319*** | 0.236** | 0.253** | 0.202* | - | 0.705*** | 0.498*** | 0.414*** | 0.405*** | 0.47*** | 0.175* | 0.228** | 0.065 | 0.163* | 0.026 |
| 1. DERS Strategies | 0.338*** | 0.323*** | 0.355*** | 0.281** | 0.705*** | - | 0.645*** | 0.339*** | 0.504*** | 0.61*** | 0.194* | 0.180* | 0.091 | 0.128 | 0.056 |
| 1. DERS Acceptance | 0.221** | 0.358*** | 0.296*** | 0.245** | 0.498*** | 0.645*** | - | 0.340*** | 0.370*** | 0.44*** | 0.181* | 0.170* | 0.062 | 0.013 | 0.059 |
| 1. DERS Awareness | 0.078 | 0.079 | 0.153 | -0.035 | 0.414*** | 0.339*** | 0.340*** | - | 0.278** | 0.02 | 0.010 | 0.085 | 0.050 | -0.064 | -0.60 |
| 1. DERS Goals | 0.006 | -0.121 | -0.131 | -0.123 | 0.405*** | 0.504*** | 0.370*** | 0.278** | - | 0.19* | -0.105 | -0.119 | -0.114 | -0.189* | -0.179* |
| 1. DERS Impulse Control | 0.34*** | 0.36*** | 0.35*** | 0.45*** | 0.47*** | 0.61*** | 0.44*** | 0.02 | 0.19* | - | 0.32*** | 0.27** | 0.18* | 0.30*** | 0.27** |
| 1. CTQ Emotional Abuse | 0.626*** | 0.583*** | 0.525*** | 0.639*** | 0.175* | 0.194* | 0.181* | 0.010 | -0.105 | 0.32*** | - | 0.833*** | 0.717*** | 0.696*** | 0.530*** |
| 1. CTQ Emotional Neglect | 0.482*** | 0.489*** | 0.482*** | 0.517*** | 0.228** | 0.180* | 0.170* | 0.085 | -0.119 | 0.27** | 0.833*** | - | 0.644*** | 0.706*** | 0.498*** |
| 1. CTQ Physical Abuse | 0.388*** | 0.410*** | 0.333*** | 0.411*** | 0.065 | 0.091 | 0.062 | 0.050 | -0.114 | 0.18* | 0.717*** | 0.644*** | - | 0.569*** | 0.437*** |
| 1. CTQ Physical Neglect | 0.461*** | 0.438*** | 0.389*** | 0.492*** | 0.163* | 0.128 | 0.013 | -0.064 | -0.189* | 0.30*** | 0.696*** | 0.706*** | 0.569*** | - | 0.549*** |
| 1. CTQ Sexual Abuse | 0.298*** | 0.408*** | 0.407*** | 0.472*** | 0.026 | 0.056 | 0.059 | -0.060 | -0.179* | 0.27** | 0.530*** | 0.498*** | 0.437*** | 0.549*** | - |

Note. This table show partial Pearson correlations between all variables of interest. * *p* < 0.05. ***p* < 0.01; ****p* < 0.001.
