# Supplementary material for: Self-reported impulsivity in women with borderline personality disorder: the role of childhood maltreatment severity and emotion regulation difficulties
Source: Borderline Personal Disord Emot Dysregul. 2019 Mar 5;6:6. doi: 10.1186/s40479-019-0101-8 (PMC6399941; doi:10.1186/s40479-019-0101-8)
Supplement: Supplementary file 6 — Conditional process analysis without the subscale Urgency. (DOCX 13 kb) [file 40479_2019_101_MOESM6_ESM.docx]

**Conditional process analysis without the subscale Urgency**

The overall regression model was significant (*F*_(7,164)_= 154.05, *p*< .0001, *R*^2^=.868), suggesting that approximately 87% of the variance in self-reported impulsivity (UPPS mean scores) was explained by all predictors in the model. Specifically, higher levels of childhood maltreatment severity (*B*=0.109, *SE*=0.014, *t*=8.04, *p<.*0001; CI: [0.082, 0.136]) and more difficulties in emotion regulation (*B*=0.023, *SE*=0.009, *t*=2.43, *p=.*016; CI: [0.004, 0.041]) predicted more impulsivity. Group, age, and education also had significant effects, with younger age and lower education being related to higher impulsivity (age: *B*=-0.036, *SE*=0.015, *t*=2.36, *p=.*019; CI: [-0.066, -0.006]; education: *B*=-0.321, *SE*=0.143, *t*=2.25, *p=.*026; CI: -[0.603, -0.039]). The effect of group was also significant (*B*=5.842, *SE*=0.589, *t*=9.93, *p<.*0001; CI: [4.679, 7.004]).

The interaction of childhood maltreatment severity and group on impulsivity was significant (*B*=0.054, *SE*=0.012, *t*=4.55, *p<.*0001; CI: [0.077, 0.030]). The interaction between group and childhood trauma in predicting DERS was not significant (*B*=0.012, *SE*=0.188, *t*=0.10, *p=.*917; CI: [-0.392, 0.352]; CTQ: *B*=0.154, *SE*=0.212, *t*=0.72, *p=.*471; CI: [-0.266, 0.573]). Based on the bootstrapping confidence interval, difficulties in emotion regulation mediated the effect of childhood trauma on impulsivity in the BPD group (*B*=0.002, *SE*=0.001, CI: [0.003, 0.005]) but not in the other groups (HC: *B*=0.003, *SE*=0.004, CI: [-0.003, 0.012]; CC: *B*=0.006, *SE*=0.002, CI: [-0.001, 0.005]).
